# Can Pallidal Deep Brain Stimulation Rescue Borderline Dystonia? Possible Coexistence of Functional (Psychogenic) and Organic Components

**DOI:** 10.3390/brainsci10090636

**Published:** 2020-09-15

**Authors:** Ryoma Morigaki, Ryosuke Miyamoto, Hideo Mure, Koji Fujita, Taku Matsuda, Yoko Yamamoto, Masahito Nakataki, Tetsuya Okahisa, Yuki Matsumoto, Kazuhisa Miyake, Nobuaki Yamamoto, Ryuji Kaji, Yasushi Takagi, Satoshi Goto

**Affiliations:** 1 Department of Advanced Brain Research, Graduate School of Biomedical Sciences, Tokushima University, Tokushima 770-8503, Japan; matsutaku.tokushima.osaka@gmail.com (T.M.); med.miyake@gmail.com (K.M.); nyamamoto@tokushima-u.ac.jp (N.Y.); 2 Department of Neurosurgery, Graduate School of Biomedical Sciences, Tokushima University, Tokushima 770-8503, Japan; hmure@tokushima-u.ac.jp (H.M.); m04079yh@jichi.ac.jp (Y.Y.); ytakagi@tokushima-u.ac.jp (Y.T.); 3 Parkinson’s Disease and Dystonia Research Center, Tokushima University Hospital, Tokushima 770-8503, Japan; ryom@tokushima-u.ac.jp (R.M.); sgoto@tokushima-u.ac.jp (S.G.); 4 Department of Neurology, Graduate School of Biomedical Sciences, Tokushima University, Tokushima 770-8503, Japan; kfujita@tokushima-u.ac.jp (K.F.); rkaji@tokushima-u.ac.jp (R.K.); 5 Division of Rehabilitation, Tokushima University Hospital, Tokushima University, Tokushima 770-8503, Japan; tetsuokahisa@tokushima-u.ac.jp; 6 Department of Psychiatry, Graduate School of Biomedical Sciences, Tokushima University, Tokushima 770-8503, Japan; nktk@tokushima-u.ac.jp; 7 Department of Radiology, Graduate School of Biomedical Sciences, Tokushima University, Tokushima 770-8503, Japan; lakaisbmtyk@gmail.com; 8 Department of Neurodegenerative Disorders Research, Graduate School of Biomedical Sciences, Tokushima University, Tokushima 770-8503, Japan

**Keywords:** dystonia disorder, movement disorders, somatoform disorder, deep brain stimulation, neuronal plasticity

## Abstract

The diagnosis and treatment of functional movement disorders are challenging for clinicians who manage patients with movement disorders. The borderline between functional and organic dystonia is often ambiguous. Patients with functional dystonia are poor responders to pallidal deep brain stimulation (DBS) and are not good candidates for DBS surgery. Thus, if patients with medically refractory dystonia have functional features, they are usually left untreated with DBS surgery. In order to investigate the outcome of functional dystonia in response to pallidal DBS surgery, we retrospectively included five patients with this condition. Their dystonia was diagnosed as organic by dystonia specialists and also as functional according to the Fahn and Williams criteria or the Gupta and Lang Proposed Revisions. Microelectrode recordings in the globus pallidus internus of all patients showed a cell-firing pattern of bursting with interburst intervals, which is considered typical of organic dystonia. Although their clinical course after DBS surgery was incongruent to organic dystonia, the outcome was good. Our results question the possibility to clearly differentiate functional dystonia from organic dystonia. We hypothesized that functional dystonia can coexist with organic dystonia, and that medically intractable dystonia with combined functional and organic features can be successfully treated by DBS surgery.

## 1. Introduction

Dystonia is a clinical syndrome diagnosed largely by clinical phenomenology [1]. Because there are so many different clinical manifestations and causes, there is no specific examination criteria or simple algorithm for diagnosing dystonia [1,2,3,4]. Dystonia is characterized by patterned, directional, sustained, or intermittent muscle contractions causing abnormal dystonic postures, and repetitive twisting dystonic movements [2,3,4,5,6]. In addition, organic dystonia patients often manifest morning benefit, overflow phenomenon, mirroring, action dystonia, and sensory trick (geste antagoniste) [4,5,6]. Morning benefit is a phenomenon where dystonic symptoms alleviate early in the morning because of relaxation or sleep [6]. Activation of dystonic movements beyond the commonly involved body regions is called overflow [4,5]. Mirror dystonia occurs in the affected region when a non-affected homologous body part performs specific tasks [5]. Action dystonia is the aggravation of dystonic movements by specific voluntary movements [4,6]. Alleviation of dystonia with tactile or proprioceptive sensory input to the affected body part is called a sensory trick; its attenuating effects may diminish with disease progression [4,5,6]. Due to the lack of specific diagnostic tests, expert observation of these manifestations is recommended in the guidelines of the European Federation of Neurological Associations [3].

The critical step in order to diagnose organic dystonia is to rule out disorders that mimic dystonia due to orthopedic, neuromuscular, or psychogenic processes [1,2,5,6]. Functional (psychogenic) dystonia is a manifestation of functional movement disorders, which are part of the wide spectrum of functional neurological disorders [7]. Functional neurological disorders account for over 16% of new patients referred to neurological clinics [8] and cause a long-term impairment in quality of life as in Parkinson’s disease [7,9]. Functional neurological symptom disorder is classified as an official term in Diagnostic and Statistical Manual of Mental Disorders (DSM)-V under the general category of somatic symptom and related disorders [10,11], and is clearly differentiated from factitious disorder and malingering [1]. Functional dystonia is the second most common condition in patients with functional movement disorders after functional tremors [12]. According to the Gupta and Lang proposed revisions, functional dystonia is diagnosed by: (a) a documented persistent relief by psychotherapy, which may also be helped by physiotherapy; (b) disappearance of movement impairment when patients believe that they are not being observed; (c) movement alterations which do not match a classical movement disorder; or (d) inconsistencies in the examination [13]. Although patients with functional fixed dystonia are considered not to exhibit overflow dystonia and sensory tricks, all positive features of organic dystonia can be observed in functional dystonia as well [14,15,16]. Therefore, diagnosis and treatment of functional dystonia is challenging for neurologists and psychiatrists [14,17,18,19]. The pathogenesis is still a mystery. Abnormal cortical and spinal excitability have been associated with functional dystonia in a few case reports [20,21]. It has been reported that there are no differences in single unit discharge characteristics during pallidal deep brain stimulation (DBS) between functional and organic dystonia [22,23]; however, the clinical course is dismal whether they receive the surgical intervention or not [22,23,24,25,26,27,28]. Because of their poor surgical outcome, patients with medically intractable functional dystonia are neglected and left untreated.

In this report, we included five patients who manifested phenomenologically with generalized- or hemi-dystonia, which was not clearly definable as organic or functional. All patients received pallidal DBS, experiencing good clinical outcomes. Our results raise important questions about the boundary between functional and organic dystonia, and about the indications for DBS surgery in patients with dystonia and functional features.

## 2. Materials and Methods

Before the surgery, written informed consent was obtained from all patients for the use of their clinical data. This study was approved by the Ethical Committee of Tokushima University Hospital (approval number 3743, date of approval 22 June 2020).

### 2.1. Subjects

We retrospectively included four generalized and one hemi-organic dystonia patients who were diagnosed as having organic dystonia by movement disorder specialists. They also manifested psychiatric features and could be simultaneously diagnosed as clinically definite functional dystonia by the Gupta and Lang Proposed Revisions.

### 2.2. Assessment Instruments

Dystonia symptoms in all patients were assessed before the surgery and at follow-up using the Burke-Fahn-Marsden Dystonia Rating Scale (BFMDRS), which includes both a movement scale and a disability scale [29]. These surgical outcomes were compared with other types of dystonia using data that we have already published [30,31]. Statistical analyses were performed using the Student’s t-test or one-way ANOVA followed by Games–Howell test. A *p* value < 0.05 was considered statistically significant.

### 2.3. Surgical Procedure

Bilateral pallidal-DBS surgery was carried out as we previously reported with some modifications [30,31]. Under general anesthesia with propofol or desflurane, semi-microelectrode recordings were performed in all patients. DBS electrodes were implanted into the bilateral ventral margins of the globus pallidus internus (GPi) and connected to the programmable pulse generators implanted subcutaneously on the same day.

## 3. Results

### 3.1. Clinical Characteristics of the Dystonia Patients

None of the patients had a family history of neurological disorders. Patients 1–4 had a history of psychiatric symptoms (Table 1) and had received medications, including benzodiazepines, tricyclic antidepressants, selective serotonin uptake inhibitors, serotonin-norepinephrine reuptake inhibitors, and anticonvulsants. Patient 4 also had a history of drug abuse with benzodiazepines and methamphetamine; therefore, she might have had tardive dystonia. The results of blood examinations evaluating the concentrations of vitamins, homocysteine, ceruloplasmin, and copper were normal; genetic screenings of DYT1 and DYT6 were negative; neuroimaging studies including brain magnetic resonance imaging/angiography were intact in all patients except Patient 5 who expressed lower levels of serum ceruloplasmin and copper. Although the ATP7B gene was negative, patient 5 was diagnosed with Wilson’s disease and was administered zinc acetate and trientine hydrochloride. His hemidystonia might have been secondary to Wilson’s disease. All patients manifested dystonic postures or movements and at least one symptom, which was characteristic of organic dystonia (Table 1). Patients 1–4 were bedridden due to severe dystonic symptoms. No one presented with parkinsonism. Medications, including intravenous injection of L-dopa, transoral administration of benzodiazepines, and trihexyphenidyl hydrochloride were not effective or only showed limited effects. Botulinum toxin injections were partially effective for Patients 1 and 5, but non-effective for the others. Baclofen trial for Patients 1 and 3 were not effective (Table 1). Before the surgery, we suspected that Patients 2 and 3 might have functional dystonia because of their history of marked alleviation of dystonia by the placebo treatment, though they displayed symptoms that we considered to be part of organic dystonia. Repetitive transcranial magnetic stimulation (rTMS) was also partially effective for Patient 2. Patients 2 and 3 were followed-up for several months; however, they were still bedridden and showed strong inclination towards DBS. Thus, we decided to perform DBS for their organic dystonia. Patients 4 and 5 experienced significant alleviation in their movement disorder when they believed themselves to be unobserved before the surgery. However, the alleviation was partial and we judged that they had predominant organic dystonia.

At the time of surgery, the mean age of the patients with dystonia was 28.2 ± 9.3 (range, 15–36) years and the mean disease duration was 3.3 ± 2.7 (range, 1.5–8.0) years. The mean preoperative BFMDRS motor score was 58.9 ± 10.7 and BFMDRS disability score was 12.8 ± 2.4.

### 3.2. Electrophysiological Findings and Postoperative Verification of the Implanted Electrodes

All patients showed a cell-firing pattern of bursting with interburst intervals during microelectrode recordings in GPi. Intraoperative X-ray system and postoperative computed tomography images demonstrated that all the electrodes were implanted in the GPi.

### 3.3. Stimulation Settings

For all patients, optimal results were obtained at the final stimulator settings with the mean current of 2.2 ± 1.6 mA on the right side (range, 1.1–4.6), and 3.2 ± 1.5 mA on the left side (range, 1.6–4.8); mean frequency of 100 ± 35.6 Hz on the right side (range, 60–180), and 84 ± 47.2 Hz on the left side (range, 20–180); and pulse width of 110 ± 66.3 μs on the right side (range, 60–200), and 194 ± 150.3 μs on the left side (range, 60–420).

### 3.4. Assessment of Symptoms after the Surgery

As shown in Table 2, the mean follow-up period after the surgery was 33.0 ± 21.4 months (range, 9–55 months); 3 of the 5 patients were followed for more than 3 years. All patients initially responded well to DBS. Patient 1 was blinded during the stimulation and showed aggravation of dystonia on the right side when left pallidal stimulation was turned off; however, no change was observed on her left side when the right pallidal stimulation was turned off. Patient 1 recovered gradually from dystonia on her left side with the right pallidal stimulation; however, 17 months after the surgery, she suddenly developed new incongruent fixed dystonia in her left hand. Patient 2 was fully recovered from her symptom and discharged. One month after full recovery and discharge, dystonia symptoms showed a sudden complete recurrence in Patient 2. Blind DBS discontinuation did not alleviate her symptoms, but she gradually recovered by placebo adjustment of the parameters and was discharged without stimulation. She had remaining mild dystonia, which appeared in the same segments as observed by placebo treatment. Two months after discharge, at the patient’s request, we restarted the stimulation and saw full recovery of the remaining dystonia. Patient 3 almost fully recovered from generalized dystonia, and almost no change except the emerging dystonic posture in her right shoulder and bilateral legs during the blind DBS discontinuation was observed. One month after the surgery, Patient 3 presented with an edema on her legs, and was diagnosed with complex regional pain syndrome, which was successfully treated with steroids. Mild recurrence of choreic movements in her hands and legs were successfully treated with placebo adjustments. Patient 4 also fully recovered from her generalized dystonia; however, she manifested very mild recurrence on her face and hands, which was successfully treated with placebo adjustments. Patient 5 showed mild response to DBS. He showed moderate alleviation when believing himself to be unobserved several times after the surgery. Blind DBS discontinuation seemed to evoke no change in his hemidystonia immediately. However, blind stimulation discontinuation for a few weeks aggravated his dystonia.

Thus, Patient 1 was diagnosed as “clinically established” and the other four patients as “documented” functional dystonia by the Fahn–Williams criteria. All patients were diagnosed with “clinically definite” functional dystonia as determined by the Gupta and Lang Proposed Revisions.

At the latest follow-up, dystonia symptoms of all patients had improved markedly. The mean BFMDRS motor score improved significantly (58.9 vs. 9.8; *p* < 0.001). The mean BFMDRS disability score also improved significantly (12.8 vs. 3.2; *p* < 0.01). Their improvement rates were 82.6 ± 22.4% (range, 63.8–100%) in motor score and 73.6 ± 37.8% (range, 18.2–100%) in disability score. Four of the five patients were no more dependent in daily life, and three of the five patients successfully got back to their work.

### 3.5. Comparison with Other Types of Dystonia

One-way ANOVA revealed that there were significant differences among three types of dystonia in age at surgery (*p* < 0.001), duration of the illness at surgery (*p* < 0.001), and preoperative BFMDRS motor score (*p* < 0.05) (Table 3).

Post-hoc analysis revealed that functional dystonia patients were significantly younger at surgery than those with tardive dystonia (*p* < 0.05), and Meige syndrome (*p* < 0.001). Patients with tardive dystonia were also younger at surgery than those with Meige syndrome (*p* < 0.01). Meige syndrome patients had significantly longer duration of the illness at surgery than patients with other type of dystonia (*p* < 0.01). The preoperative BFMDRS motor score was significantly worse in functional dystonia patients compared to Meige syndrome patients (*p* < 0.05). There was no difference among three types of dystonia in follow-up periods, preoperative BFMDRS disability score, postoperative BFMDRS motor and disability scores, and improvement rate in BFMDRS motor and disability scores. Thus, the improvement in BFMDRS scores by pallidal DBS was the same among the three groups.

## 4. Discussion

Historically, Oppenheim’s dystonia was considered to be a hysteric reaction before the discovery of the DYT1 gene [7,32]. Focal task specific dystonia (FTSD) was thought to be psychogenic in origin until the 1980s. Idiopathic dystonia often develops after mental stress as in hereditary dystonia DYT11 (DYT-SGCE) and DYT8 (paroxysmal nonkinesigenic dyskinesia) [32,33,34]. Psychiatric comorbidities are often discussed in dystonia patients [34]. Thus, it seems that dystonia is often very close to the mental and psychiatric symptoms. Our five patients were defined as clinically established or definite functional dystonia according to the Fahn–Williams criteria or the Gupta and Lang Proposed Revisions [13,35]. Although dystonia specialists argued carefully about the symptoms before the surgery, and judged some of these symptoms as organic, we could not clearly find the borderline between functional and organic dystonia. Our patients manifested a typical cell-firing pattern of organic dystonia patients that was observed in other studies [22,23] and the clinical outcomes were the same as those of other types of organic dystonia. Two important issues that should be clarified are whether functional dystonia can be clearly differentiated from organic dystonia, and whether dystonia patients with functional features can be excluded from surgical intervention.

Fixed abnormal posture is considered a typical manifestation of functional dystonia [7]. Given that FTSD arises from the over-practice of specific tasks, it is not surprising that doing the same fixed abnormal posture might cause abnormal firing in the brain motor circuitry. Currently, there is no firing pattern data from normal human GPi or thalamus. The data from patients with Parkinson’s disease and dystonia, or from non-human primates, suggests that long-term suppression in patients with dystonia typically shows abnormal neuronal activity [36,37,38,39,40,41]. Kobayashi et al. and Ramos et al. described that thalamic, as well as GPi activities, observed in the intraoperative microelectrode recordings in patients with functional dystonia exhibit a cell-firing pattern of bursting with interburst intervals, and this is similar to the cell-firing pattern of organic dystonia [22,23]. We also detected a cell-firing pattern of bursting with interburst intervals in all patients [42,43]. Thus, it is plausible that an abnormal posture induces a cell-firing pattern of bursting with interburst intervals.

In addition to the performance of specific tasks, it is suggested that genetic predisposition underlies the cause of FTSD [44]. There also exists the maladaptive plasticity in sensorimotor integration in patients with FTSD [44]. Espay et al. reported that suppression of cortical neurons and subsequent suppression of spinal neurons by TMS paired stimulations were abnormal in both patients with functional and organic dystonia [21]. This means that changes in cortical plasticity are present both in patients with functional and organic dystonia. Although genetic predisposition or epigenetic changes might be required, prolonged fixation in an abnormal posture in patients with functional dystonia might generate secondary organic dystonia and make up complexity of functional and organic dystonia [45]. In contrast, primary existing organic dystonia in our patients might have intermingled with subsequent functional dystonia. Functional dystonia has been reported to co-occur with other organic movement disorders (functional overlay) [7,12,16,46]; however, our study focuses on the co-existence of functional and organic components in a single movement disorder, i.e., dystonia.

Increasing evidence support the notion that functional movement disorders are caused by malfunction in brain networks, making it difficult to differentiate organic from functional dystonia [1,11]. Abnormal connectivity between cognitive, limbic, and motor neural networks seems to occur in patients with functional movement disorders [11]. The impairments in the hub structures of limbic and motor circuits, such as the striosome compartment in the striatum, might be important for its pathogenesis [47]. It might be important to continue modulating the neural network in order to stop primary organic dystonia or to prevent establishment of a vicious circle induced by secondary organic dystonia. We conclude that we should not deny the application of the surgical intervention to dystonia patients just because they are diagnosed as functional by the Fahn–Williams criteria or Gupta and Lang Proposed Revisions. If there is an organic component, DBS surgery might help alleviate dystonia. The development of new criteria for surgical intervention in dystonia patients with functional features is needed.

## Figures and Tables

**Table 1:** Characteristics of patients with borderline dystonia who underwent bilateral pallidal deep brain stimulation.

	Patient 1	Patient 2	Patient 3	Patient 4	Patient 5	Mean ± SD
Age (yr) at the surgery	15	33	35	22	36	28.2 ± 9.3
Sex	F	F	F	F	M	-
Age at onset (yr)	13	25	32	20	34	24.8 ± 8.6
Duration of disease (yr)	2	8	3	2	1.5	3.3 ± 2.7
Type of dystonia	Generalized	Generalized	Generalized	Generalized	Hemidystonia	-
Core dystonic feature	a	a, b	a, b	b	a	-
Other symptoms related to the organic dystonia	c	c, d	c, e	c, e, f	c, e	-
Fahn-Williams criteria	Clinically established	Documented	Documented	Documented	Documented	-
Gupta and Lang proposed revisions	Clinically definite	Clinically definite	Clinically definite	Clinically definite	Clinically definite	-
Diagnosed psychiatric disturbance	BPD	Panic disorder, DD	DD	BPD	None	-
Therapies received before (upper column) and after (lower column) DBS	M, BTX, R, PT	M, BTX, rTMS, R	M, BTX, baclofen (trial), R	M, BTX, R, PT	M, BTX, R	
	M, BTX, baclofen (trial), R, PT, OSSCS	M, R	M, R	M, R, PT	M, BTX, R	

SD: standard deviation, a: dystonic posture, b: dystonic movement, c: action dystonia, d: morning benefit, e: mirroring, f: overflow, BPD: borderline personality disorder, DD: dissociative disorder, DBS: deep brain stimulation, M: medications, BTX: botulinum toxin, R: rehabilitation, PT: psychotherapy, OSSCS: orthopedic selective spasticity-control surgery, rTMS: repetitive transcranial magnetic stimulation.

**Table 2:** Detailed information about pallidal deep brain stimulation in patients with borderline dystonia.

	Patient 1	Patient 2	Patient 3	Patient 4	Patient 5	Mean ± SD
Follow-up after surgery (months)	55	52	36	13	9	33.0 ± 21.4
Implanted devices system	Model 3387 Medtronic Activa SC	4 contacts leads Abbott Brio	4 contacts leads Abbott Brio	Directional leads BS Vercise Gevia	Directional lead BS Vercise Gevia	-
Stimulation parameters						
Electrode right/left	2+1-/2+1- bipolar	2+1-/2+1- bipolar	C+2-/C+2- monopolar	C+1-2-3-4-/C+1-2-3-4- monopolar	NA/C+3-4-5-6- monopolar	-
Current (mA) right/left	1.5/3.1	1.1/1.6	2.3/1.9	4.6/4.6	-/4.8	2.2 ± 1.6/3.2 ± 1.5
Pulse width (μS) right/left	120/420	200/250	200/150	60/60	/180	110 ± 66.3/194 ± 150.3
Frequency (Hz) right/left	80/80	60/60	180/180	130/130	/20	100 ± 35.6/84 ± 47.2
BFMDRS-M (max = 120)						
Preoperatively	70	68.5	59.5	51	45.5	58.9 ± 10.7
Postoperatively	26	0	1	1	21	9.8 ± 12.6
Percent improvement (%)	62.9	100	98.3	98.0	53.8	82.6 ± 22.4
BFMDRS-D (max = 30)						
Preoperatively	14	16	13	10	11	12.8 ± 2.4
Postoperatively	7	0	0	0	9	3.2 ± 4.4
Percent improvement (%)	50.0	100	100	100	18.2	73.6 ± 37.8

SD: standard deviation, NA: not available, BS: Boston Scientific co., BFMDRS-M, -D: Burke-Fahn-Marsden Dystonia Rating Scale-motor score, -disability score.

**Table 3:** Comparison of patients with functional dystonia, tardive dystonia, and Meige syndrome.

Type of Dystonia	Age (yr)	Duration (yr)	BFMDRS-M (max = 120), Mean ± S.D.			BFMDRS-D (max = 30) Mean ± S.D.		
			Preoperatively	Postoperatively	Percent Improvement (%)	Preoperatively	Postoperatively	Percent Improvement (%)
Functional dystonia (*n* = 5)	28.2 ± 9.3 *^,^^††^	3.3 ± 2.7 ^††^	58.9 ± 10.7 ^†^	9.8 ± 12.6	82.6 ± 22.4	12.8 ± 2.4	3.2 ± 4.4	73.6 ± 37.8
Tardive dystonia [30] (*n* = 6)	44.5 ± 8.7	3.1 ± 2.2 ^††^	30.8 ± 22.7	3.8 ± 3.8	85.5 ± 14.4	9.3 ± 4.8	1.8 ± 1.7	80.2 ± 12.2
Meige syndrome [31] (*n* = 5)	64.6 ± 7.2	12.4 ± 4.2	22.2 ± 12.4	3.1 ± 1.7	84.2 ± 6.8	11.2 ± 7.9	1.4 ± 1.5	89.4 ± 8.0

BFMDRS-M or -D: Burke-Fahn-Marsden dystonia rating score-motor scale or -disability scale. * *p* < 0.05 vs. Tardive dystonia, ^†^ *p* < 0.05, ^††^ *p* < 0.01 vs. Meige syndrome. One-way ANOVA followed by the Games-Howell test was used.
